# The influence of the *PRKAG3 *mutation on glycogen, enzyme activities and fibre types in different skeletal muscles of exercise trained pigs

**DOI:** 10.1186/1751-0147-53-20

**Published:** 2011-03-24

**Authors:** Anna Granlund, Marianne Jensen-Waern, Birgitta Essén-Gustavsson

**Affiliations:** 1 Department of Clinical Sciences, Section for Comparative Physiology and Medicine, Faculty of Veterinary Medicine and Animal Science, Swedish University of Agricultural Sciences, SE-750 07, Uppsala, Sweden

## Abstract

**Background:**

AMP-activated protein kinase (AMPK) plays an important role in the regulation of glucose and lipid metabolism in skeletal muscle. Many pigs of Hampshire origin have a naturally occurring dominant mutation in the AMPK γ3 subunit. Pigs carrying this *PRKAG3 *(R225Q) mutation have, compared to non-carriers, higher muscle glycogen levels and increased oxidative capacity in *m. longissimus dorsi*, containing mainly type II glycolytic fibres. These metabolic changes resemble those seen when muscles adapt to an increased physical activity level. The aim was to stimulate AMPK by exercise training and study the influence of the *PRKAG3 *mutation on metabolic and fibre characteristics not only in *m. longissimus dorsi*, but also in other muscles with different functions.

**Methods:**

Eight pigs, with the *PRKAG3 *mutation, and eight pigs without the mutation were exercise trained on a treadmill. One week after the training period muscle samples were obtained after euthanisation from *m. biceps femoris*, *m. longissimus dorsi, m. masseter *and *m. semitendinosus*. Glycogen content was analysed in all these muscles. Enzyme activities were analysed on *m. biceps femoris*, *m. longissimus dorsi*, and *m. semitendinosus *to evaluate the capacity for phosphorylation of glucose and the oxidative and glycolytic capacity. Fibre types were identified with the myosin ATPase method and in *m. biceps femoris *and *m. longissimus dorsi*, immunohistochemical methods were also used.

**Results:**

The carriers of the *PRKAG3 *mutation had compared to the non-carriers higher muscle glycogen content, increased capacity for phosphorylation of glucose, increased oxidative and decreased glycolytic capacity in *m. longissimus dorsi *and increased phosphorylase activity in *m. biceps femoris *and *m. longissimus dorsi*. No differences between genotypes were seen when fibre type composition was evaluated with the myosin ATPase method. Immunohistochemical methods showed that the carriers compared to the non-carriers had a higher percentage of type II fibres stained with the antibody identifying type IIA and IIX fibres in *m. longissimus dorsi *and a lower percentage of type IIB fibres in both *m. biceps femoris *and *m. longissimus dorsi*. In these muscles the relative area of type IIB fibres was lower in carriers than in non-carriers.

**Conclusions:**

In exercise-trained pigs, the *PRKAG3 *mutation influences muscle characteristics and promotes an oxidative phenotype to a varying degree among muscles with different functions.

## Background

The prevalence of the *PRKAG3 *mutation in RN^- ^Hampshire pigs has likely been propagated by its favourable effects on the growth rate and on the meat content of the carcass [1,2]. This *PRKAG3 *mutation is a substitution in the *PRKAG3 *gene (R225Q), which encodes a muscle specific isoform of the AMP-activated protein kinase (AMPK) γ3 subunit expressed mainly in glycolytic muscles in pigs [3,4]. AMPK is an energy sensor that is activated by an increase in AMP/ATP ratio and directly phosphorylates many metabolic enzymes and therefore plays an important role in glucose uptake, glycogen synthesis, and fat oxidation in skeletal muscle [5,6]. AMPK activation by muscle contraction is a vital step towards exercise-stimulated glucose uptake [7,8]. Glycogen will repeatedly be broken down and resynthesised when a muscle is trained which leads to a demand for glucose uptake and activation of AMPK to restore the glycogen used during exercise. Pigs that carry the *PRKAG3 *mutation have in comparison to non-carriers greater glycogen content and increased oxidative capacity in *m. longissimus dorsi *[4,9]. These metabolic changes resemble those seen in pigs when muscles have adapted to an increased physical activity level [10,11]. Few studies have looked into the effect of the *PRKAG3 *mutations on other skeletal muscles than *m. longissimus dorsi*. Different muscles have different functions within the body, which is reflected by different metabolic and contractile properties of their muscle fibres. For example *m. masseter *is a muscle that is mainly active during the chewing process and *m. biceps femoris *seems to be a muscle that is more active than *m. semitendinosus *and *m. longissimus dorsi*, when pigs are trained on a treadmill [10,11]. Contractile characteristics based on different myosin heavy chain (MHC) isoforms differ among fibres and muscles [12]. Hybrid fibres contain more than one MHC isoform and may indicate fibre type transformation. An increased amount of hybrid fibres can be seen in trained muscles of man and rat [13]. The aim of this study was to examine the effect of the *PRKAG3 *mutation on both the metabolic profile and the fibre characteristics in different muscles (*m. longissimus dorsi*, *m. biceps femoris, m. semitendinosus *and *m. masseter*) after exercise-induced stimulation of AMPK and glycogen metabolism.

## Methods

### Animals and housing

The Ethical Committee for Animal Experiments, Uppsala, Sweden approved of the experimental design.

Sixteen clinically healthy female pigs (Yorkshire/Swedish Landrace × Hampshire) at the age of 9-11 weeks and with a mean weight of 29 ± 0.6 kg were obtained from the University herd. Eight pigs were heterozygous carriers and eight pigs were non-carriers of the *PRKAG3 *mutation which was revealed by DNA analyses of blood [3]. All pigs were housed at the department (Department of Clinical Sciences, Swedish University of Agricultural Sciences) in pens with concrete floors and straw as bedding. The animals were fed twice daily *ad libitum *a commercial finisher diet without growth promoters (Piggfor; Origio 522 PK, Lantmännen, Sweden with an energy content of 12.4 MJ and crude protein content of 13%), and had *ad libitum *access to water. Clinical health examinations were performed daily on all animals throughout the study.

### Experimental design

The protocol ran for nine weeks and started with a two week period of acclimatisation. During this period the pigs also became used to the treadmill (Säto, Knivsta, Sweden). They were allowed to walk and trot on the treadmill for a few minutes on four separate days, before an exercise test was performed and tissue samples from *m. biceps femoris *were obtained by a needle biopsy [14]. Thereafter the pigs trained on the treadmill once daily, five days a week for the next five weeks. The speed continuously increased from 1.5 m/s to 2.5 m/s and the distance increased from 300 m to 1000 m. The training period ended with a second exercise test and tissue samples were again obtained from *m. biceps femoris*. Thereafter the pigs had a jugular catheter inserted under general anaesthesia to obtain unstressed blood samples. Also a catheter in situ facilitated a smooth euthanisation and muscle samples were achieved under a minimum of stress. A third exercise test was then performed a week later and tissue samples from *m. biceps femoris *as well as blood samples were obtained. The pigs were then 18 to 20 weeks old and the carriers had a mean weight of 80 ± 1.5 kg and the non-carriers had a mean weight 74 ± 3 kg with no significant difference between the two genotypes.

Six days after the third exercise test the animals were euthanised by an intravenous infusion of pentobarbital (100 mg/mL) in their pens. Two pigs were withdrawn from the study after training, one due to unwillingness to run on the treadmill and the other did not survive anaesthesia.

### Muscle samples

Within 10 min after the animals were euthanised, samples of about 2 × 1 × 1 cm were taken from *m. masseter*, *m. semitendinosus *(white portion), *m. biceps femoris *and *m. longissimus dorsi *(caudal to the last rib) by excision. All muscle specimens were obtained from the centre of the middle part of the muscle. The tissue samples were immediately frozen in liquid nitrogen and stored at minus 80°C until analysed. The tissue sample used for histochemistry was rolled in talcum powder before being frozen.

### Muscle fibre analyses

**Figure 1:**
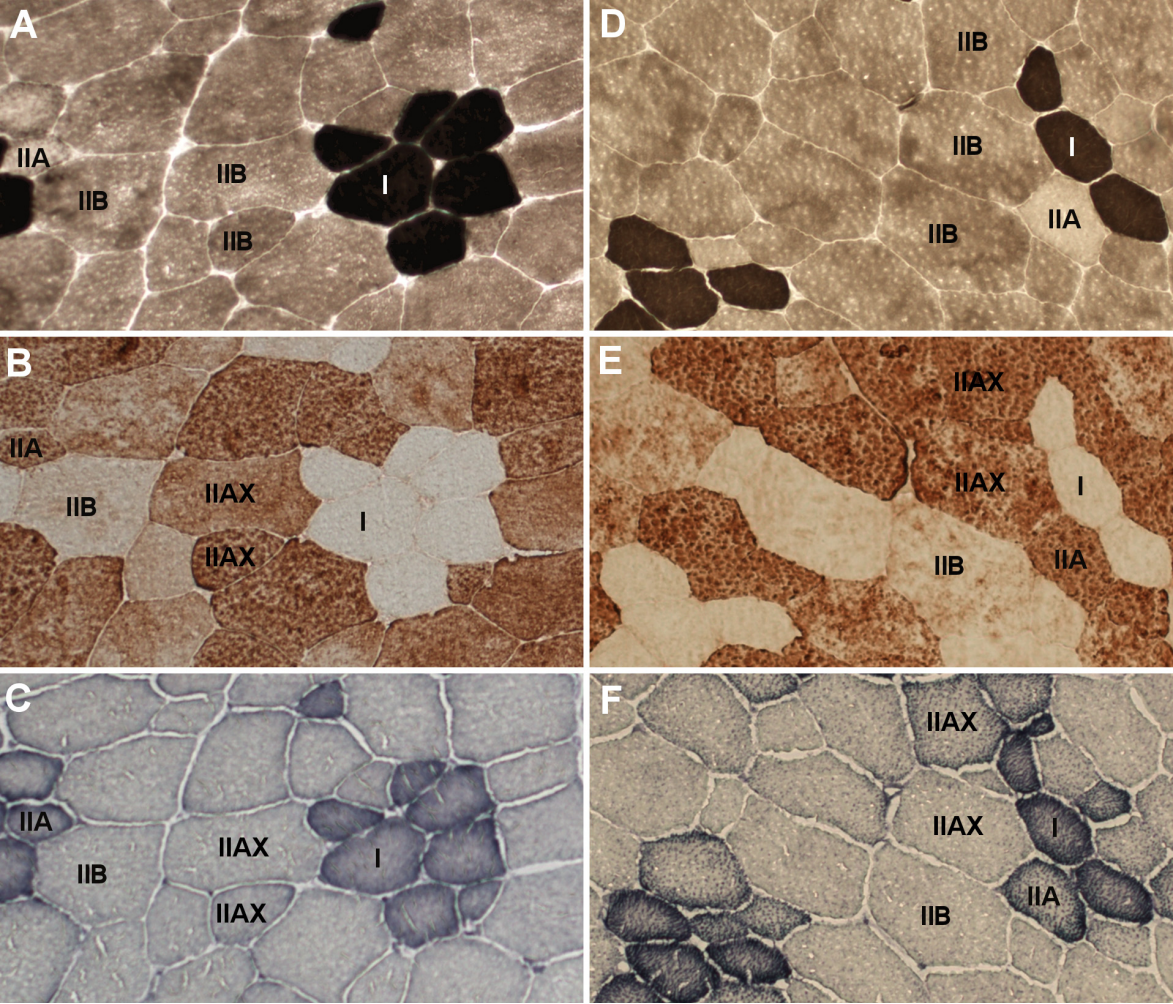
**Photomicrographs of serial sections of *m. longissimus *dorsi of carriers (A, B, C) and non-carriers (D, E, F) of the *PRKAG3 *mutation**. Fibre types I, IIA, and IIB classified with myosin ATPase (pH 4.6) stains (A, D) and fibre type IIAX is classified with immunohistochemical (A4-74) stains (B, E). Note that many type IIB fibres in the myosin ATPase stain were classified as IIAX fibres with the immunohistochemical stain and that some of these IIAX fibres may be pure, IIX or IIBX fibres. Oxidative capacity is evaluated from the NADH tetrazolium reductase stains (C, F). Note that type I fibres have a high staining intensity, whereas staining intensity varies among the subgroups of type II fibres.

The muscle sample was mounted on embedding medium (OCT compound) and serial transverse sections (10 μm) were cut in a cryostat (2800 Frigocut E, Reichert-Jung, Leica Microsystems GmbH) at -20°C. Myofibrillar ATPase staining with preincubations at pH 4.3, 4.6 and 10.3 were used to identify fibre types I, IIA, IIB [15] in all muscles. In *m. biceps femoris *and *m. longissimus dorsi *also immunohistochemical methods were used. Serial sections, were reacted with myosin heavy chain (MHC) antibodies BA-D5 (MHCI) (gift from E.Barrey) and A4-74 (MHCIIA + MHCIIX) (Alexis Biochemicals). The secondary antibody (rabbit anti-mouse immunoglobulins) and the peroxidase-anti-peroxidase complex used to visualize the binding to the antibody came from Dako in Denmark. The muscle fibres stained with the antibody A4-74, were classified as IIAX fibres and some of these fibres may be pure IIX and/or IIBX fibres. To evaluate fibre type composition, fibre type area and relative fibre type area, a computerized image analyser (Bio-Rad, Scan Beam, Hadsund, Denmark) was used. One section (containing at least 200 fibres) of the pH 4.6 ATPase stain were photographed and type IIB fibres on this section that corresponded to fibres that stained with the A4-74 antibody were classified as type IIAX fibres. All type I fibres from the ATPase stain corresponded to type I fibres stained with the antibody BA-D5 (MHCI). Sections of *m. biceps femoris *and *m. longissimus dorsi *were also stained with the NADH tetrazolium reductase method [16]. Oxidative capacity was subjectively evaluated from the intensity of the blue staining (30-50 fibres of each type) into high- if the whole fibre was stained, medium- if some staining was apparent, mostly at the cells borders, or low if there was hardly any staining within the cell (Figure 1).

### Enzyme activity analyses

Muscle biopsies were freeze-dried overnight and then muscle tissue was dissected out under a microscope to remove visible blood, fat, and connective tissue. To determine the activities of citrate synthase (CS), 3-hydroxyacyl-CoA dehydrogenase (HAD), lactate dehydrogenase (LDH), hexokinase (HK), and phosphorylase, 1-2 mg of pure tissue was homogenized with an ultrasound disintegrator (Branson) in ice-chilled potassium phosphate buffer (0.1 M, pH 7.3) at a dilution of 1:400 and then analysed fluorometrically [14,17].

### Glycogen analyses

For glycogen determination 1-2 mg of pure tissue was boiled in 1 M HCl for 2 h to form glucose residues. Glucose was analysed with a fluorometric method [17].

### Statistical analyses

Data are presented as means ± standard errors. For the statistical analyses the values from each genotype were assumed to be independent observations from normal probability distributions. An unpaired t-test was used for comparison of values between the carrier and the non-carrier pigs. Means were regarded as significantly different at *P *< 0.05. Statistical analyses were carried out using Sigma Stat Statistical Software version 11.0.

## Results

### Fibre type composition and mean fibre area

None of *m. masseter*, *m. biceps femoris, m. semitendinosus *or *m. longissimus dorsi *showed any difference between genotypes in the percentage of type I, IIA and IIB fibres when evaluated from the ATPase stains. Type IIB fibres from the ATPase stain for *m. longissimus dorsi *and *m. biceps femoris *correspond to the sum of IIAX and IIB fibres identified with the immunohistochemical method. A large proportion of type IIB fibres identified from the ATPase stain was seen in *m. semitendinosus*, *m. longissimus dorsi *and *m. biceps femoris*. *M. semitendinosus *and *m. longissimus dorsi *had a low proportion of type I fibres. A high proportion of type IIA fibres were seen in *m. masseter *(Table 1, 2).

**Table 1:** Fibre characteristics in different muscle groups in carriers and non-carriers of the *PRKAG3 *mutation.

	*m. biceps femoris*		*m. longissimus dorsi*	
	
	Carriers (n = 7)	Non-carriers (n = 7)	Carriers (n = 7)	Non-carriers (n = 7)
Fibre type (%)				
I	27 ± 1	24 ± 1	15 ± 1	13 ± 1
IIA	7 ± 1	8 ± 1	4 ± 1	2 ± 1
IIAX	37 ± 2	33 ± 2	56 ± 3 *	38 ± 3
IIB	29 ± 1 *	35 ± 1	25 ± 2 *	47 ± 3
Fibre area (μm^2^)				
I	2471 ± 194 *	1896 ± 146	2571 ± 196 *	1965 ± 192
IIA	3346 ± 330 *	2424 ± 173	2118 ± 326	1949 ± 426
IIAX	5443 ± 399 *	3352 ± 276	5088 ± 430 *	3856 ± 354
IIB	7054 ± 592 *	5224 ± 500	4630 ± 396	4576 ± 229
Relative fibre area (%)				
I	15 ± 2	14 ± 1	9 ± 1	6 ± 1
IIA	5 ± 1	6 ± 1	2 ± 1	2 ± 1
IIAX	41 ± 2 *	33 ± 2	65 ± 3 *	39 ± 3
IIB	39 ± 3 *	47 ± 1	25 ± 3 *	53 ± 4
NADH intensity (%)				
I High	100 ± 0	100 ± 0	100 ± 0	100 ± 0
IIA High	12 ± 6	12 ± 5	17 ± 17	0 ± 0
IIA Medium	88 ± 6	88 ± 5	83 ± 17	100 ± 0
IIAX Medium	57 ± 4 *	92 ± 3	25 ± 4 *	61 ± 6
IIAX Low	43 ± 4 *	8 ± 3	75 ± 4 *	39 ± 6
IIB Medium	2 ± 1	2 ± 1	13 ± 3 *	4 ± 2
IIB Low	98 ± 1	98 ± 1	87 ± 3 *	96 ± 2

Fibre type composition was identified with myosin ATPase stains and type I and IIA+ IIX with myosin heavy chain antibodies.

NADH-tetrazolium reductase staining intensity was subjectively evaluated as low, medium and high in the different fibre types.

Data as means ± SE. * *P *< 0.05 significantly different to non-carriers.

The immunohistochemical method showed that pigs carrying the *PRKAG3 *mutation had compared to the non-carriers less (*P *< 0.05) percentage of type IIB fibres, in *m. biceps femoris *and in *m. longssimus dorsi *and a higher (*P *< 0.05) percentage of type IIAX fibres in *m. longissimus *dorsi. The mean fibre area of all different fibres types in the carriers was larger (*P *< 0.05) in *m. biceps femoris *and larger (*P *< 0.05) in type I and type IIAX fibres in *m. longissimus *dorsi. In these muscles the relative area of type IIAX fibres was larger (*P *< 0.05) in the carriers and the relative area of type IIB fibres was lower (*P *< 0.05) than in the non-carriers (Table 1).

In all type I fibres the NADH staining intensity was high (Figure 1). Most of the type IIA fibres were stained medium while type IIAX and IIB fibres stained both medium and low in *m. biceps femoris *and *m. longissimus dorsi*. Most real type IIB fibres stained low in both muscles The carriers had, in both *m. biceps femoris *and *m. longissimus dorsi*, a lower percentage (*P *< 0.05) of medium stained type IIAX fibres and a higher (*P *< 0.05) percentage of low stained type IIAX fibres compared to the non-carriers. The staining intensity in type IIB fibres was mainly low, but the carriers had a higher (*P *< 0.05) percentage of medium stained type IIB fibres and a lower (*P *< 0.05) percentage of low stained type IIB fibres in *m. longissimus dorsi *(Table 1).

There were no genotype differences in fibre type area and relative fibre type area in *m. semitendinosus *and *m. masseter *(Table 2).

**Table 2:** Fibre characteristics in different muscle groups in carriers and non-carriers of the *PRKAG3 *mutation.

	*m. semitendinosus*		*m. masseter*	
	
	Carriers (n = 7)	Non-carriers (n = 6)	Carriers (n = 7)	Non-carriers (n = 7)
Fibre type (%)				
I	16 ± 1	13 ± 4	28 ± 4	32 ± 4
IIA	4 ± 1	6 ± 1	70 ± 3	67 ± 4
IIB	80 ± 1	81 ± 4	2 ± 0	1 ± 1
Fibre area (μm^2^)				
I	2388 ± 118	2386 ± 228	1659 ± 186	2142 ± 235
IIA	2574 ± 402	2905 ± 380	2127 ± 302	2264 ± 266
IIB	4574 ± 285	4504 ± 446	1429 ± 175	2192 ± 181
Relative fibre area (%)				
I	9 ± 1	8 ± 1	24 ± 4	31 ± 4
IIA	3 ± 1	4 ± 0	74 ± 4	68 ± 4
IIB	88 ± 1	88 ± 1	1 ± 0	2 ± 1

Fibre type composition was identified using myosin ATPase stains.

Data as means ± SE.

### Enzyme activities

The CS, HAD, LDH, HK and phosphorylase activities of *m. longissimus dorsi*, *m. biceps femoris*, and *m. semitendinosus *in the carriers and the non-carriers of the *PRKAG3 *mutation are presented in Table 3. The CS activity was higher (*P *< 0.05) in the carriers of the *PRKAG3 *mutation than in the non-carriers only in *m. longissimus dorsi*, and there was no difference between genotypes regarding HAD activity in any of the muscles. The activity of LDH was lower (*P *< 0.05) in the carriers of the *PRKAG3 *mutation in *m. longissimus dorsi *and in *m. semitendinosus *than in the non-carriers. In all muscles the activity of HK was higher (*P *< 0.05) in the carriers and the activity of phosphorylase was higher (*P *< 0.05) in *m. biceps femoris *and *m. longissimus dorsi *in the carriers than in the non-carriers.

**Table 3:** Enzyme activities and glycogen concentration in different muscle groups in carriers and non-carriers of the *PRKAG3 *mutation.

	*m. longissimus dorsi*		*m. semitendinosus*		*m. biceps femoris*	
	
	Carriers (n = 6)	Non-carriers (n = 6)	Carriers (n = 6)	Non-carriers (n = 7)	Carriers (n = 7)	Non-carriers (n = 7)
CS	20 ± 3 *	8 ± 5	15 ± 1	13 ± 2	19 ± 2	20 ± 1
HAD	24 ± 1	24 ± 2	26 ± 2	26 ± 2	31 ± 3	29 ± 3
HK	8 ± 1 *	3 ± 2	7 ± 1 *	4 ± 1	8 ± 1 *	5 ± 1
Phosphorylase	18 ± 2 *	15 ± 2	17 ± 2	15 ± 3	16 ± 2 *	11 ± 1
LDH	2778 ± 328 *	3199 ±134	2929 ± 187 *	3255 ± 203	2474 ± 219	2561 ± 179
Glycogen	725 ± 46 *	458 ± 32	600 ± 49 *	349 ± 18	681 ± 42 *	420 ± 28

Data are expressed as mmol/kg/min for citrate synthase (CS), 3-hydroxyacyl-CoA (HAD), hexokinase (HK), phosphorylase, lactate dehydrogenase (LDH) and in mmol/kg for glycogen concentration.

Data as means ± SE. * *P *< 0.05 significantly different from non-carriers.

### Glycogen analyses

Pigs carrying the *PRKAG3 *mutation had in *m. longissimus dorsi*, *m. biceps femoris and m. semitendinosus *a higher (*P *< 0.05) concentration of glycogen (Table 3) than the non-carriers. In *m. masseter *the glycogen concentration was also higher (*P *< 0.05) in the carriers (268 ± 26 mmol/kg) than in the non-carriers (166 ± 19 mmol/kg).

## Discussion

The main new finding of this study is, that after exercise training the *PRKAG3 *mutation influences metabolic and fibre characteristics to a varying degree among muscles with different functions. Fibre type composition and the physical activity level of the muscle are factors that may contribute to the differences seen in glycogen content and enzyme activities between muscles. In agreement with earlier studies on untrained pigs, the pigs carrying the *PRKAG3 *mutation had in comparison to the non-carriers, higher content of glycogen in both *m. longissimus dorsi *and in *m. biceps femoris *[1,18,19]. Previous studies have shown that the mutation does mainly affect white glycolytic muscles such as *m. longissimus dorsi *and has no effect on a red muscle such as *m. semispinalis capitis *[4]. *M. masseter *is considered to be a red muscle based on a high CS activity and low glycolytic potential whereas *m.longissimus *is a glycolytic muscle based on a low CS activity and high glycolytic potential [20]. *M. semitendinosus *of non-carriers had similar metabolic and fibre characteristics as seen in *m. longissimus dorsi *and is thus considered to be a white glycolytic muscle. As expected the carriers of the *PRKAG3 *mutation had higher glycogen content also in this muscle. The fact that the total glycogen content seemed to be somewhat lower in *m. semitendinosus *than in *m. longissimus dorsi *is in agreement with earlier observations of non-carriers of the *PRKAG3 *mutation [10]. Notable was that the carriers of the *PRKAG3 *mutation had higher glycogen content than the non-carriers also in *m. masseter*, which is considered to be a red oxidative muscle. However, as seen in the present study, some glycolytic type II fibres exist in this muscle. These may be influenced by the mutation, resulting in overall higher glycogen content. The higher synthesis of glycogen in the muscles of the carriers of the *PRKAG3 *mutation is likely related to a higher capacity for phosphorylation of glucose as indicated by the higher HK activity observed in the muscles. The *PRKAG3 *mutation may also have an effect on glycogenolysis in association with high muscle glycogen storage as indicated by the higher phosphorylase activity found in both *m. longissimus dorsi *and *m. biceps femoris *in the carriers. The higher phosphorylase and HK activity observed in *m. biceps femoris *of the exercise trained carriers is in agreement with results on young untrained carriers [14]. This indicates that the *PRKAG3 *mutation has a great influence on these enzymes and may suggest that the carriers of the mutation have an increased glycogen turnover. The increased oxidative capacity (indicated by the higher CS activity) and the decreased glycolytic capacity (indicated by lower LDH activity) in *m. longissimus dorsi *of the carriers of the *PRKAG3 *mutation, is also in agreement with earlier studies of untrained pigs [4,9]. In a previous study the HAD activity was higher in *m.longissimus dorsi *[4] but this was not seen in any of the muscles in the present study. A study with transgenic mice models showed that mice with a chronically AMPK-activating mutation caused a shift from fibre type B to IIA/X fibres [21]. These mice had higher activity of CS and increased hexokinase protein expression regardless if they had exercised or not. AMPK signalling was suggested to play an important role for transforming skeletal muscle fibre types as well as for increasing hexokinase II protein expression and oxidative capacity. These findings are in agreement with effects of the *PRKAG3 *mutation on muscle characteristics in the present study especially in *m. longissimus dorsi*. Studies on transgenic mice (Tg-Prkag3^225Q^) have shown that the *PRKAG*3 mutation is associated with a greater basal AMPK activity [22]. Previous studies of fibre characteristics in *m. longissimus dorsi *in pigs that carry the *PRKAG3 *mutation indicate that alterations may occur in the subgroups of type II fibres [4,23]. This is also in agreement with the findings of the present study. Notable was that the carriers of the *PRKAG3 *mutation had less IIB fibres, not only in *m. longissimus dorsi*, but also in *m. biceps femoris*, compared to non-carriers. The fact that the oxidative capacity evaluated by the CS activity in the present study did not differ between genotypes in *m. biceps femoris *but differed in *m. longissimus dorsi*, may be related to these muscles being differently involved during locomotion [10]. It has earlier been indicated that adaptations to training differ between muscles [10]. Endurance trained pigs had in comparison to non-trained pigs an increased oxidative capacity and a higher glycogen content in *m. biceps femoris*, but no differences were seen in *m. longissimus dorsi *and in *m. semitendinosus*, muscles thus considered to be less involved during training on a treadmill [10].

In both genotypes training adaptations in the fibres of *m. biceps femoris *may have caused a similar oxidative capacity in response to the increased energy demand during locomotion. A previous study of pigs has shown that glycogen is lowered in both genotypes in type I, IIA and in some IIB fibres in *m. biceps femoris *during the same type of exercise as used in this study, which indicates that these fibres have been recruited [19]. Adaptations to exercise training in this muscle may have decreased the effects of the *PRKAG3 *mutation on muscle metabolic and contractile properties.

The carriers had less type IIB fibres in *m. longissimus dorsi *which indicates that one effect of the *PRKAG3 *mutation may be associated with transformation of type IIB towards type IIX and IIA fibres, as carriers also had more type IIAX fibres. The muscle fibres that are classified as MHCIIAX may be a mixture of pure IIX and/or hybrid IIA+IIX and IIX+IIB as the antibody A4-74 identifies both IIA and IIX fibres [24,25]. Transition of myosin heavy chains is said to follow a sequential, yet reversible, pathway: I↔IIA↔IIAX↔IIX↔IIB [26,27]. Interestingly, genetic selection for growth performance in pigs, shifts fibre type towards type IIB fibres [28,29] whereas endurance exercise training has been shown to shift the fibre type towards type IIA in rats [30] and in man [31]. Studies in pigs also indicate that fibre type shifts from type IIB to IIA may occur with training [32,33]. Oxidative capacity is known to increase with training and among fibre types oxidative metabolism is high in type I fibres and decreases in the rank order from type I to type IIA to type IIX to type IIB fibres [34]. Intensive selection for a higher meat content and lean muscle growth in modern pigs has not only caused shifts in contractile fibre types, but also induced a change in muscle metabolism towards a more glycolytic and less oxidative fibre type [35]. In contrast, the *PRKAG3 *mutation has been shown to decrease IIB and increase IIA and IIX mRNA expression, which also implies that the genotype promotes a more oxidative phenotype [23]. The changes seen in muscle characteristics in the carriers with the *PRKAG3 *mutation thus resemble those seen when muscles in pigs adapt to an increased physical activity level. In rabbits contractile activity induces a fast-to-slow and glycolytic-to-oxidative fibre transition in skeletal muscle [36]. In the present study the pigs with the mutation in the γ-subunit of AMPK seem to have developed a more oxidative phenotype independent of contractile activity. This is supported by the higher CS activity and the higher oxidative capacity of type IIB muscle fibre types according to the NADH-tetrazolium reductase staining found in *m. longissimus dorsi *of the carriers. Notable, many type IIAX fibres in the carriers were classified as having a low oxidative capacity. However, these IIAX fibres in the carriers probably also had an overall higher oxidative capacity as they were larger in size. As seen from Figure 1, the staining intensity for NADH-tetrazolium reductase is usually homogeneous within a fibre, but more intense at the periphery due to a higher density of mitochondria there.

The muscle fibre composition of *m. masseter, m. semitendinosus*, *m. biceps femoris *and *m. longissimus dorsi *identified according to the ATPase stains is in good agreement with earlier studies [10,37]. If differences among subgroups of type II fibres (including hybrids) also occurred in *m. semitendinosus *and *m. masseter *between the two genotypes is not known, since only the ATPase staining technique was used to identify fibre types in these muscles. If fibre types had been identified only from the ATPase stains in *m. longissimus dorsi *and *m. biceps femoris*, no changes in subgroups of type II fibres between the two genotypes would have been revealed. This clearly shows that the use of antibodies against the different myosin heavy chains will give a more detailed picture of the fibre type composition in a muscle. When pure IIX and hybrid IIA+IIX and IIX+IIB fibres cannot be detected alterations in muscle fibre types might be overlooked.

The MHCIIB isoform was previously said to exist only in small animals such as mouse, rat, guinea pig and rabbit [38,12]. However, studies have shown that large animals i.e. pigs and llamas do exhibit MHCIIB fibres and mostly in glycolytic muscles [34,39,40]. In fact the *m. longissimus dorsi *has been shown to contain 51% of type MHCIIB in pigs [41]. This is in good agreement with 47% type IIB fibres observed in the *m. longissimus dorsi *of non-carriers in the present study. As seen from Figure 1 the NADH-staining intensity showed marked differences in oxidative capacity among the fibre types and as expected type IIB fibres had mainly a low oxidative capacity. Whether type IIAX fibres with low staining intensity for oxidative capacity correspond to pure type IIX and/or hybrid type IIX + IIB needs to be investigated in future studies using antibodies that can separate MHCIIA and MHCIIX fibres.

## Conclusions

In exercise-trained pigs, the *PRKAG3 *mutation influences muscle characteristics and promotes an oxidative phenotype to a varying degree among muscles with different functions. The present results show that the carriers of the *PRKAG3 *mutation are of interest not only in meat science, but also as a large animal model for *in vivo *studies of the carbohydrate metabolism.

## Competing interests

The authors declare that they have no competing interests.

## Authors' contributions

All authors participated in the design of the study and the collection of samples. AG performed laboratory analyses and statistical calculations. AG and BEG have interpreted the data and drafted the manuscript. All authors read and approved the final manuscript.
